# Dienogest vs. combined oral contraceptive: A systematic review and meta‐analysis of efficacy and side effects to inform evidence‐based guidelines

**DOI:** 10.1111/aogs.15145

**Published:** 2025-05-01

**Authors:** Ilaria Piacenti, Veronica Tius, Maria Federica Viscardi, Anna Biasioli, Martina Arcieri, Stefano Restaino, Ludovico Muzii, Giuseppe Vizzielli, Maria Grazia Porpora

**Affiliations:** ^1^ Department of Obstetrics and Gynecology “Santa Maria” Hospital Terni Italy; ^2^ Department of Medicine University of Udine Udine Italy; ^3^ Department of Maternal, Infantile and Urological Sciences University of Rome La Sapienza Rome Italy; ^4^ Clinic of Obstetrics and Gynecology, “S. Maria Della Misericordia” University Hospital, Azienda Sanitaria Universitaria Friuli Centrale (ASUFC) Udine Italy; ^5^ Biomedical Sciences, Gender Medicine, Child and Women Health University of Sassari Sassari Italy

**Keywords:** combined oral contraceptives, dienogest, dyspareunia, efficacy, endometriosis, hormonal treatment, pelvic pain, safety, tolerability

## Abstract

**Introduction:**

Dienogest is a synthetic fourth‐generation progestin that has been approved for the medical treatment of endometriosis, and its efficacy on pain symptoms and quality of life is well established even in the long term. Nowadays, only a few controlled trials evaluating the safety of dienogest compared with other hormonal therapies have been published. This systematic review and meta‐analysis aims to compare efficacy and tolerability data between dienogest and combined oral contraceptives (COC) in patients taking hormonal therapy for endometriosis treatment in order to inform evidence‐based guidelines.

**Material and Methods:**

PubMed (Medline), Web of Science, and Google Scholar were systematically searched from the inception of each database until October 2024. Selection criteria included any articles comparing efficacy outcomes and at least one tolerability data between dienogest and COC in patients diagnosed with endometriosis. Studies comparing COC containing Dienogest or another type of hormonal treatment were excluded. A random‐effects meta‐analysis was conducted if adequate data were available from at least three studies, reporting pooled mean differences and odds ratios between groups using Review Manager V.7.9.0. PROSPERO registration number: CRD42024598455.

**Results:**

A total of four randomized control trials and one observational study were included, showing moderate risk at bias assessment. Meta‐analysis did not show any statistical difference in improving pelvic pain after treatment [CI 95% (−1.45–1.17); *I* ^2^ = 86%; *p* = 0.84]. In contrast, dyspareunia after treatment was significantly lower in the COC group [CI 95% (0.64–1.33); *I* ^2^ = 0%; *p* < 0.00001]. No statistical difference was found in terms of vaginal bleeding [OR = 0.88; CI 95% (0.39–1.96); *I* ^2^ = 41%; *p* = 0.75], nausea and vomiting [OR = 0.51; CI 95% (0.16–1.63); *I* ^2^ = 67%; *p* = 0.26], headache [OR = 0.91; CI 95% (0.38–2.21); *I* ^2^ = 59%; *p* = 0.84], hot flushes [OR = 1.16; CI 95% (0.54–2.48); *I* ^2^ = 0%; *p* = 0.71], and hair loss [OR = 1.69; CI 95% (0.52–5.53); *I* ^2^ = 46%; *p* = 0.39]. Treatment discontinuation rate was similar between groups.

**Conclusions:**

Dienogest is comparable to COC in terms of efficacy and tolerability. The therapeutic choice should be based on the patient's preference, clinical history, and experience.

## Graphical abstract

Dienogest is comparable to combined oral contraceptives in terms of expected efficacy and tolerability profiles, showing no higher incidence rates of side effects such as irregular vaginal bleeding, nausea and vomiting, headache, hot flashes, and hair loss.

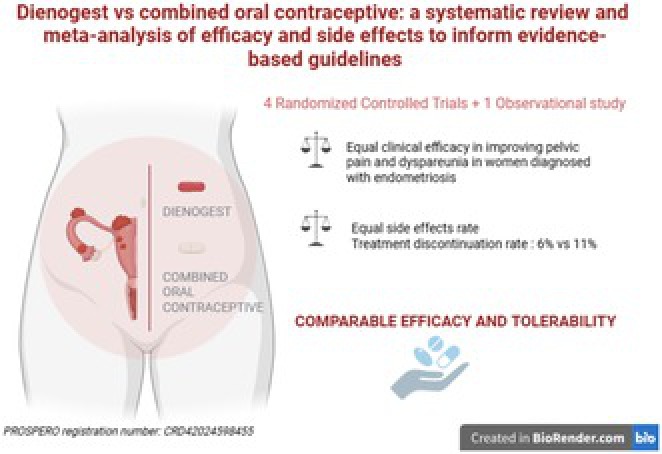

**Abbreviations**
CI: confidence interval
COC: combined oral contraceptives
DNG: dienogest
GnRH: gonadotropin hormone‐releasing hormone
OR: odds ratio
RCT: randomized controlled trial
ROBINS‐I: Risk of Bias In Non‐randomizedrandomised Studies of Interventions
VAS: visual analog scale

**Key message**

Compared with combined oral contraceptives, dienogest has an equal clinical efficacy and similar tolerability profile in patients taking hormonal therapy for endometriosis.

## 1 INTRODUCTION

Endometriosis is a chronic inflammatory condition, estrogen‐dependent, and affects up to 10% of women of reproductive age. It is characterized by endometrial‐like tissue outside the uterus.^1^ This condition typically presents with dysmenorrhea (painful menstruation), dyspareunia (pain during intercourse), persistent pelvic pain, and infertility, reducing the quality of life.^2^, ^3^, ^4^ The management of endometriosis should be personalized for each individual, considering factors, such as the severity of symptoms, types of lesions, and reproductive goals. Guidelines recommend surgical, pharmacologic, and nonpharmacologic approaches for treating patients with endometriosis, indicating pharmacologic treatment (hormonal and non‐hormonal therapy) as the first‐line therapeutic option.^5^ This condition often requires long‐term management strategies to minimize the need for repeated surgeries.^6^ The purpose of hormonal therapy is to block the hypothalamic–pituitary–ovarian axis, inducing amenorrhea and reducing the progression of the disease. The most common drugs include progestins and combined oral contraceptives (COC), which are effective on symptoms and are considered the most suitable options for long‐term therapy.^7^, ^8^, ^9^, ^10^ Dienogest (DNG) is a synthetic fourth‐generation progestin that has been approved for the medical treatment of endometriosis^11^; its efficacy on pain symptoms and quality of life is well established even in the long term.^12^ Nowadays, only a few controlled trials evaluating the safety of DNG compared with other hormonal therapies have been published. The aim of this systematic review and meta‐analysis is to summarize the most recent data on side effects and adverse events associated with DNG compared with COC, focusing on the frequency of reported events during its use. Additionally, the second aim of this study is to evaluate the current evidence of the efficacy of DNG vs. COC.

## 2 MATERIAL AND METHODS

### 2.1 Search strategy

A systematic search of PubMed (MEDLINE) and Google Scholar was conducted on 07 August 2024. The search strategy was performed using combinations of the following MeSH terms: “dienogest”, “tolerability,” “side effects,” “safety,” and “endometriosis.” The articles published from 1948 to 2024 were found in the literature and were uploaded onto the Rayyan platform. This systematic review and meta‐analysis were reported following the Preferred Reporting Items for Systematic Reviews and Meta‐analyses (PRISMA) guidelines,^13^ and it has been registered in the international prospective register of systematic reviews (PROSPERO) (Registration number: CRD42024598455).

### 2.2 Eligibility criteria and study selection

We included studies with the following inclusion criteria: (1) studies enrolling adult women diagnosed with endometriosis undergoing oral hormonal treatment, (2) studies involving patients diagnosed with endometriosis comparing efficacy outcomes and safety between treatment with DNG and treatment with COC, and (3) studies reporting data about compliance, side effects, and treatment discontinuation rate.

We excluded non‐human studies (animal and in vitro), studies enrolling patients diagnosed with adenomyosis (alone or concomitant to endometriosis), and studies comparing COC containing DNG or another type of hormonal treatment (e.g., gonadotropin‐releasing hormone agonists, gonadotropin‐releasing hormone antagonists, intrauterine device‐releasing levonorgestrel, and danazol).

The primary outcome was the comparison of tolerability between groups, defined by qualitative and quantitative analysis of side effects and the number of patients dropping out of the treatment before the end of the observation. The secondary outcome was the comparison of treatments' efficacy in improving pelvic pain, dysmenorrhea, and/or dyspareunia. Outcome eligibility required reporting at least one of the primary outcomes of interest.

Published studies and unpublished material were subjected to the same rigorous methodological evaluation. Single case reports, congress proceedings, meta‐analyses, book chapters, and editorial letters were excluded. There were no language or publication date restrictions. Three reviewers (TV, PI, VMF) independently reviewed and screened titles and abstracts according to the predefined strategy and criteria. Any article identified as having the potential to fulfill our inclusion criteria underwent full‐text evaluation. The authors discussed discrepancies in decisions regarding study inclusion until agreement was reached.

### 2.3 Data extraction

Microsoft Excel was used to collect and summarize data. Data extraction was carried out by three authors simultaneously (TV, PI, VMF). The following data and information were extracted for each study, where available: first author, year of publication, country of origin, study design, number of participants, interventions, comparisons, and length of treatment (weeks). Characteristics of enrolled patients were also collected; for example, endometriosis stage; presence and intensity of pelvic pain, dysmenorrhea, and dyspareunia at baseline, measured using the visual analog scale (VAS) or as a percentage of patients reporting severe symptoms and previous medical treatment. Then, efficacy outcomes were extracted, defined by the VAS value of pelvic pain, dysmenorrhea, and dyspareunia after the treatment period or the percentage of patients reporting the persistence of severe symptoms. Lastly, compliance and tolerability data (treatment discontinuation rate due to side effects and type and percentage of side effects related to treatment) were collected.

### 2.4 Quality assessment

Two independent reviewers (PI, VMF) assessed the risk of bias of the included studies. The quality of the included studies was evaluated using the ROB2 tool^14^ for randomized studies and the Risk of Bias In Non‐randomized Studies of Interventions (ROBINS‐I) scale^15^ for observational studies.

### 2.5 Statistical analysis

Meta‐analysis was performed by one author (TV) using the generic inverse‐variance method with a random‐effects model, and it was conducted if adequate data were available from at least three studies. Mean difference with 95% confidence intervals (CI) was calculated for continuous variables, while odds ratio (OR) with 95% two‐sided confidence intervals (CI) was calculated for categorical variables. The heterogeneity of the included articles was assessed with the I‐square (I^2^) tool, with a value <25% being considered low heterogeneity and >75% being regarded as high heterogeneity. Review Manager Web software, version 7.9.0 (The Nordic Cochrane Center, The Cochrane Collaboration, Copenhagen, Denmark) was used to analyze the data, and a *p*‐value below 0.05 was considered statistically significant.

## 3 RESULTS

### 3.1 Study selection

The search strategy uncovered a total of 4315 articles. Once duplicates (204 articles) were eliminated, 4111 unique articles were screened by reviewing titles and abstracts in order to identify those that met the inclusion criteria. Full‐text copies of the remaining 9 articles were obtained and analyzed again for eligibility. After that, four articles^16^, ^17^, ^18^, ^19^ were excluded as they did not provide data of interest according to the main objectives of our study.

**FIGURE 1:**
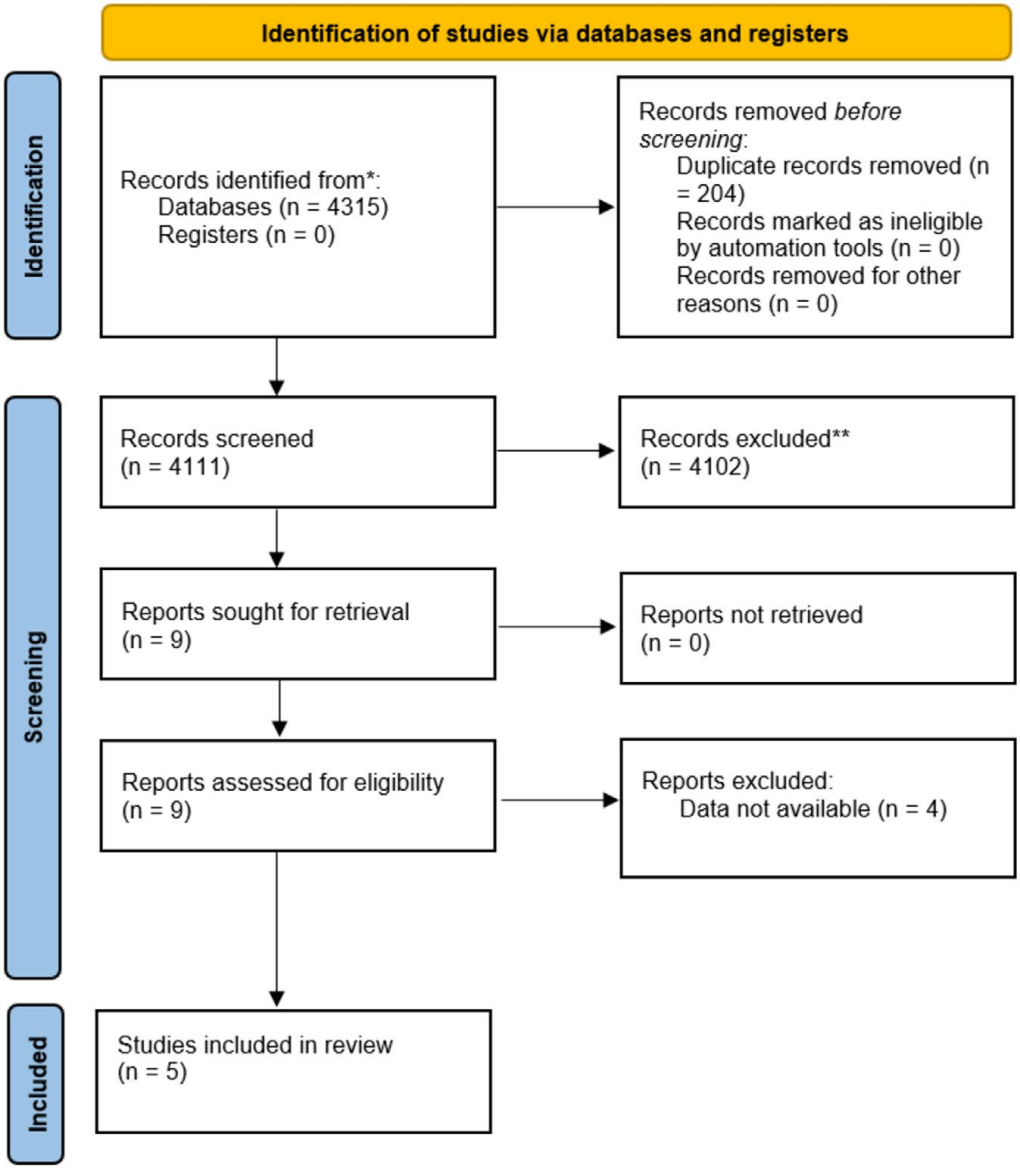
PRISMA flowchart of article selection phases.

Final analysis incorporated a total of 5 studies: 4 randomized controlled trials (RCT)^20^, ^21^, ^22^, ^23^, ^24^ and one observational study,^24^ comprising a total of 459 patients (Figure 1). Of the total number of patients, 191 were given DNG (Group 1) while 268 patients received COC (Group 2); two studies^20^, ^21^ used ethinylestradiol/drospirenone and three studies^22^, ^23^, ^24^ used ethinylestradiol/levonorgestrel as comparator drugs (Table S1).

### 3.2 Quality assessment

Regarding the randomization process, the four included RCTs did not show a high overall risk of bias, as well as performance and detection bias. Most of the RCTs showed an “unclear” risk of attrition bias, except for one^21^ that was considered a high risk. RCTs bias analyses are detailed in Figure S1. The included observational study^24^ received an “unclear” bias rating, according to the ROBINS‐I scale, due to concerns about confounding variables (Figure S2).

### 3.3 Baseline patient characteristics

Table S2 summarizes the baseline characteristics of enrolled patients. Incorporated studies included patients diagnosed with endometriosis regardless of stage of disease, except two studies^22^, ^23^ that focused exclusively on advanced and most severe stages of endometriosis (III–IV). Regarding pelvic pain at baseline, no statistical difference was found between comparison groups (*p* = 0.13). Equally, baseline VAS values of dyspareunia were not found to be statistically different among the included studies reporting this data (*p* = 0.08). Only one study^24^ reported dysmenorrhea VAS value at baseline. Two studies^20^, ^24^ reported data on the number of patients who had previously been treated with other medical drugs: 42.9% and 69.8% in the DNG group and 28.6% and 88.4% in the COC group, respectively. The average length of the treatment period of included studies was 27.2 weeks.

### 3.4 Effect of DNG on pain improvement

**FIGURE 2:**
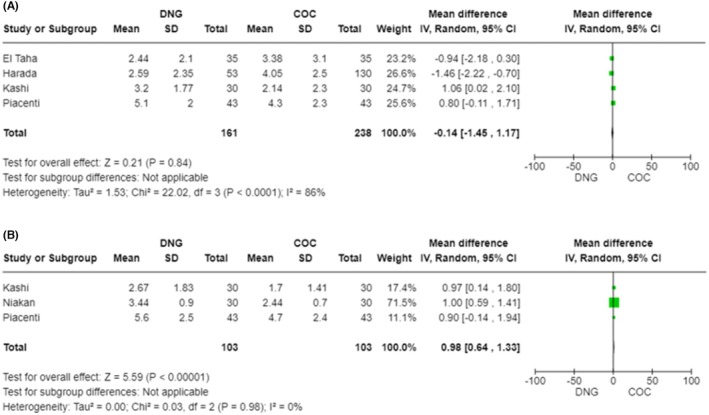
(A) Forest plot of pelvic pain after treatment. (B) Forest plot of dyspareunia after treatment.

A random‐effect meta‐analysis was performed between groups of comparison in terms of efficacy outcomes, reported in Table S3. Figure 2 shows a forest plot of pelvic pain and dyspareunia after treatment with DNG or COC.

Regarding the VAS value of pelvic pain after the treatment, statistical analysis showed a pooled mean difference of −0.14 VAS value [CI 95% (−1.45–1.17); *I* ^2^ = 86%; *p* = 0.84]. We found high heterogeneity; however, sensitivity analysis was not performed due to limited evidence. As shown in Figure 2B, the VAS value of dyspareunia after treatment was statistically significantly lower in the COC group than in the DNG group, with a pooled mean difference of 0.98 VAS value [CI 95% (0.64–1.33); *I* ^2^ = 0%; *p* < 0.00001]. Meta‐analysis of dysmenorrhea after treatment was not performed due to paucity of data.

### 3.5 Side effects and tolerability of DNG

**FIGURE 3:**
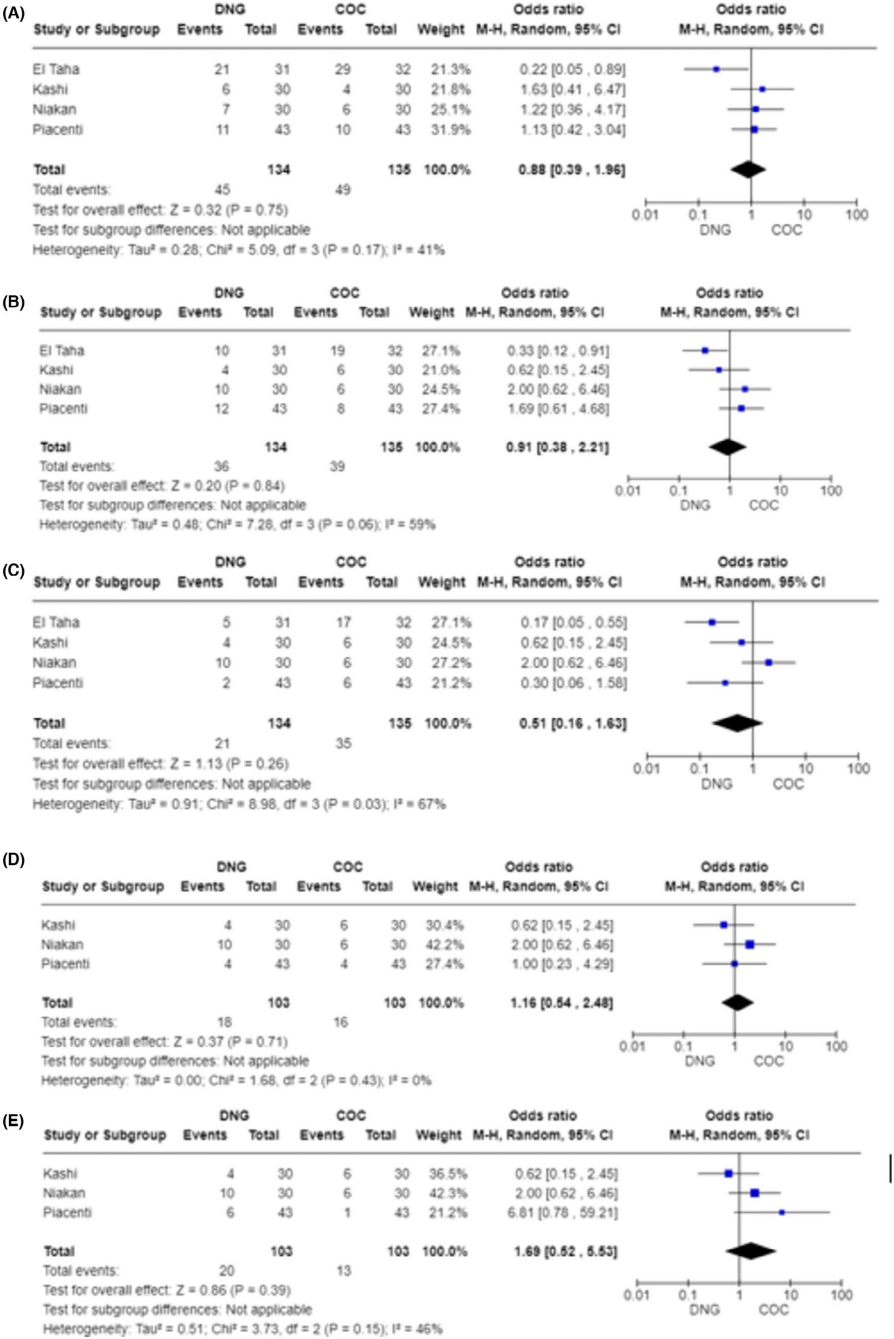
(A) Forest plot of vaginal bleeding. (B) Forest plot of nausea and vomiting. (C) Forest plot of headache. (D) Forest plot of hot flushes. (E) Forest plot of hair loss.

All included studies reported tolerability data, as given in Table S4. Meta‐analysis was performed when the side effect was reported from at least three studies. In this context, no statistical difference was found in terms of: vaginal bleeding [OR = 0.88; CI 95% (0.39–1.96); *I* ^2^ = 41%; *p* = 0.75], nausea and vomiting [OR = 0.51; CI 95% (0.16–1.63); *I* ^2^ = 67%; *p* = 0.26], headache [OR = 0.91; CI 95% (0.38–2.21); *I* ^2^ = 59%; *p* = 0.84], hot flushes [OR = 1.16; CI 95% (0.54–2.48); *I* ^2^ = 0%; *p* = 0.71] and hair loss [OR = 1.69; CI 95% (0.52–5.53); *I* ^2^ = 46%; *p* = 0.39] (Figure 3).

**FIGURE 4:**
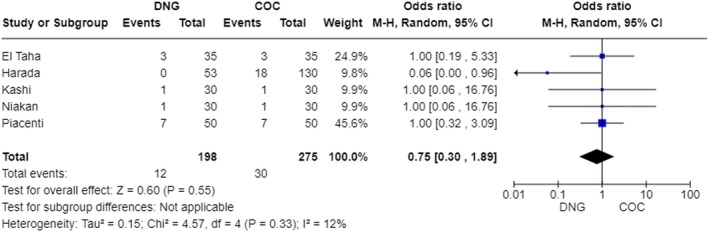
Forest plot of total drop out.

The treatment discontinuation rate due to intolerable side effects was 6% in the DNG group and 10.9% in the COC group, with no significant difference (*p* = 0.55) (Figure 4). Two studies specified reasons for treatment dropout: El Taha^20^ indicated irritability and weight gain as the most frequent causes of discontinuation in both groups of comparison; Piacenti^24^ found vaginal bleeding in the DNG group and the need for surgery and the desire for pregnancy in the COC group as the primary reasons for dropout, respectively.

## 4 DISCUSSION

The results showed no significant differences in tolerability profile, suggesting that the safety profile of DNG is comparable to that of different COCs. The data indicate that the most frequently reported side effects, such as vaginal bleeding, nausea, vomiting, headache, hot flashes, and hair loss, occurred with a similar frequency among the groups treated with DNG and those using COCs. This suggests that patients using DNG may not be at greater risk of side effects compared with those using COCs. Regarding our secondary outcome, data extracted from our meta‐analysis do not show any significant difference in efficacy between DNG and COCs, except for dyspareunia; however, this difference was not clinically relevant. Moreover, there is a remarkable decrease in the intensity of deep dyspareunia in both treatment groups.

This meta‐analysis has the following strengths: (1) comparison of efficacy outcomes and tolerability data between DNG and COC, which are both first‐line hormonal treatments for endometriosis,^6^ (2) we included as comparator drugs exclusively COC not containing DNG to assure unbiased results, (3) low or moderate heterogeneity of most data, and (4) most included studies were randomized clinical trials. Nevertheless, this meta‐analysis shows several limitations, such as (1) limited availability of data of interest in all included studies, which resulted in working with a small number of patients, (2) most of the included studies had a moderate risk of bias, and (3) average treatment duration was slightly more than 6 months; therefore, information on long‐term tolerability and safety of treatment was limited.

Our finding aligns with the recent literature; La Torre et al. conducted a cohort study involving 114 patients with endometriosis undergoing long‐term treatment with DNG for 36 months. Their findings confirm the safety of DNG and suggest it is a compelling long‐term treatment for all phenotypes of endometriosis. The study notes that, although the side effects are minimal, treatment discontinuation primarily occurs within the first year.^25^ Harada et al. conducted a 24‐week comparison of the efficacy and safety of DNG and buserelin acetate.^26^ They found that DNG was comparable to buserelin acetate in alleviating pain and other symptoms of endometriosis and enhancing quality of life. However, DNG was associated with a higher frequency of irregular genital bleeding and fewer instances of hot flushes compared with buserelin acetate. Notably, the reduction in bone mineral density (BMD) during DNG treatment was significantly lower than that observed with buserelin acetate. In terms of bone mineral density, literature describes the negative effects of long‐term DNG treatment, which may limit its long‐term use, particularly for young women and adolescents who have not reached maximum bone density. However, the extent of its impact on BMD is no greater than that of gonadotropin hormone‐releasing hormone (GnRH) agonist, LNG‐IUS, and buserelin acetate according to recent systematic review.^6^ Further studies on the long‐term use of DNG (beyond 1 year) will be necessary to definitively assess its effect on bone mineral density. Additionally, Momoeda et al. assessed the safety and efficacy of DNG over 52 weeks in 135 Japanese patients with endometriosis, further supporting its use in clinical practice.^27^

The lack of significant differences in the side effect profile carries important clinical implications. Healthcare professionals may consider DNG as a valid and well‐tolerated therapeutic option for patients needing hormonal therapy, particularly in clinical contexts where patients may have concerns about side effects. Continuously monitoring tolerability and effectiveness over time is essential to ensure proper patient management. In our meta‐analysis, we have highlighted and primarily studied and speculated on the most common reported side effects. However, other reported side effects which might be relevant, hence worth investigating, include mood swings and depression, which in some patients may have been the cause of dropout, even if they are quite infrequent.

In conclusion, this meta‐analysis suggests that DNG presents a side effect profile comparable to COCs, facilitating its adoption in clinical practice since women may perceive it as a safe choice.

The literature on the effects of DNG and oral contraceptives on endometriosis‐associated pain is extensive, but few comparative studies are available. DNG and COCs are the most studied medications, due to their low cost, good tolerability, and proven efficacy in managing endometriosis and its associated symptoms.^28^, ^29^ DNG stands out in its class due to several distinguishing features, including the absence of systemic androgenic activity, good oral bioavailability, and the same effectiveness in controlling endometriosis‐associated pelvic pain compared with GnRH agonists.^30^, ^31^, ^32^, ^33^ In addition, DNG showed potent antiproliferative effects on eutopic and ectopic endometrial tissue, independent of estrogen levels. Regarding COCs, included articles considered those containing drospirenone or levonorgestrel. Growing evidence suggests that drospirenone may be effective in reducing dysmenorrhea in patients with endometriosis, making it an attractive option as a progestin‐only or combined treatment for these patients. Additionally, drospirenone has antimineralocorticoid effects, and the absence of fluid retention enhances patient compliance.^34^, ^35^ Similarly, levonorgestrel can help manage symptoms by suppressing the growth of endometrial tissue and reducing menstrual bleeding. Its anti‐inflammatory properties may also aid in alleviating the associated pain. Additionally, levonorgestrel/ethinylestradiol seems to be less associated with venous thrombotic events than other progestogen‐containing COC.^36^

An RCT by Harada et al. demonstrated that ethinylestradiol 20 mcg/drospirenone 3 mg significantly reduces endometriosis‐associated pain compared with a placebo. However, its efficacy overlaps with DNG's.^21^ Similarly, El Taha et al. report that both COCs and DNG are effective on pain, well tolerated, and safe on medium‐term administration, significantly reducing the intensity of cerebral perfusion pressure and dyspareunia.^20^ These data are supported by studies assessing individually the efficacy of DNG and COCs and by those reporting the non‐inferiority of DNG vs. GnRH agonists.^37^, ^38^ The efficacy in pain relief appears as early as the first few months of treatment and is sustained over the long term.^24^

In patients taking DNG, pain control seems to be maintained even after discontinuing therapy.^24^ Several studies address how long pain relief from DNG persists after discontinuation, but specific durations vary among individuals.^25^ Various factors, including the patient's unique circumstances and the duration of the initial treatment, can affect the exact time frame for maintaining efficacy on pain post‐treatment. According to Niakan et al., there is a considerable impact of pain on the quality of life, which also negatively impacts both relationships and sexuality.^23^ In conclusion, DNG and COCs are effective treatments for endometriosis due to their ability to suppress ovarian function, control uterine bleeding, and reduce inflammation. Both options provide a long‐term approach to improving the quality of life for patients suffering from this condition, making them valuable therapeutic alternatives.^38^

These findings hold significant value for patients, suggesting that treatment options can be tailored to individual needs and preferences rather than being only related to their effectiveness in relieving symptoms. DNG may be recommended for those who prefer a progestin‐only option, while COC may be more appropriate for patients requiring a broader approach.

We await the VIPOS study's results, a prospective, non‐interventional, active surveillance study evaluating the safety of DNG and other hormonal treatments for the management of endometriosis conducted over 7 years in six European countries (Germany, Poland, Russia, Hungary, Switzerland, and Ukraine) to confirm or refute our findings.^16^, ^17^

## 5 CONCLUSION

The principal findings of our meta‐analysis suggest that DNG is comparable to COCs in terms of expected efficacy and tolerability profiles, showing no higher incidence rates of side effects such as irregular vaginal bleeding, nausea and vomiting, headache, hot flashes, and hair loss. Consequently, the choice of DNG over COC in clinical practice must be tailored to the patient's preferences and reported feedback during the treatment period.

## AUTHOR CONTRIBUTIONS

**Ilaria Piacenti:** Conceptualization, supervision, writing—original draft preparation. Veronica Tius: writing—original draft preparation, supervision. **Maria Federica Viscardi:** writing—original draft preparation. **Anna Biasioli:** supervision. **Martina Arcieri** and **Stefano Restaino:** supervision, project administration. **Ludovico Muzii, Giuseppe Vizzielli**, and **Maria Grazia Porpora:** Conceptualization, supervision, project administration. All authors have read and agreed to the published version of the manuscript.

## CONFLICT OF INTEREST STATEMENT

The authors declare no conflict of interest.

## Supporting information


Figures S1–S2.

Tables S1–S4.

## Data Availability

All data relevant to the study are included in the article or uploaded as supplementary information. All data were extracted from previously published studies; thus, they are publicly available.
