# Supplementary material for: Dienogest vs. combined oral contraceptive: A systematic review and meta‐analysis of efficacy and side effects to inform evidence‐based guidelines
Source: Acta Obstet Gynecol Scand. 2025 May 1;104(8):1424–32. doi: 10.1111/aogs.15145 (PMC12283171; doi:10.1111/aogs.15145)
Supplement: Supplementary file 1 — Figures S1–S2. [file AOGS-104-1424-s001.docx]

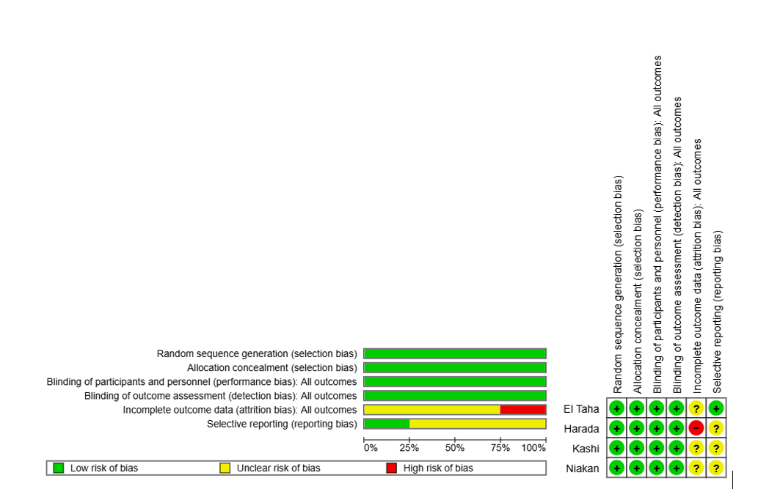
**Figure S1. ROB1 for randomized controlled trials summary and graph.**


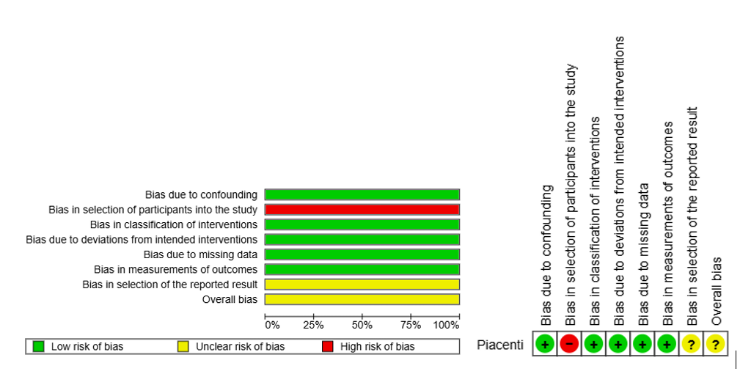
**Figure S2. ROBINS-I for observational study summary and graph.**
