# Supplementary material for: Dienogest vs. combined oral contraceptive: A systematic review and meta‐analysis of efficacy and side effects to inform evidence‐based guidelines
Source: Acta Obstet Gynecol Scand. 2025 May 1;104(8):1424–32. doi: 10.1111/aogs.15145 (PMC12283171; doi:10.1111/aogs.15145)
Supplement: Supplementary file 2 — Tables S1–S4. [file AOGS-104-1424-s002.docx]

**Table S1. General features of included studies**

| Study (Ref) | Year | Country | Type of study | Total number of patients enrolled | | Study drug | Comparator |
| --- | --- | --- | --- | --- | --- | --- | --- |
|  |  |  |  | DNG | COC |  |  |
| El Taha (20) | 2021 | Lebanon | RCT | 35 | 35 | DNG 2 mg | EE/DRSP |
| Harada (21) | 2017 | Japan | RCT | 53 | 130 | DNG 2 mg | EE/DRSP |
| Kashi (22) | 2021 | Iran | RCT | 30 | 30 | DNG 2 mg | EE/LNG |
| Niakan (23) | 2021 | Iran | RCT | 30 | 30 | DNG 2 mg | EE/LNG |
| Piacenti (24) | 2021 | Italy | Prospective cohort | 43 | 43 | DNG 2 mg | EE/LNG |

RCT = Randomized Controlled Trial; DNG = Dienogest; COC = Combined Oral Contraceptives; DRSP = Drospirenone; LNG = Levonorgestrel

**Table S2. General characteristics of enrolled patients**

|  | Previous medical treatment | Endometriosis stage | Pelvic Pain at baseline* | | Dysmenorrhea at baseline* | | Dyspareunia at baseline* | | Treatment period (weeks) |
| --- | --- | --- | --- | --- | --- | --- | --- | --- | --- |
|  |  |  | DNG | COC | DNG | COC | DNG | COC |  |
| El Taha^20^ | Yes (DNG 42.9% vs COC 28.6%) | I-IV | 8.3 ± 1.4 | 7.9 ± 1.5 | NA | NA | NA | NA | 24 |
| Harada^21^ | NA | I-IV | 7.6 ± 1.6 | 7.7 ± 1.6 | NA | NA | NA | NA | 52 |
| Kashi^22^ | NA | IV | 8.5 ± 2.2 | 7.9 ± 3.2 | NA | NA | 4.8 ± 0.8 | 4.5 ± 1.2 | 24 |
| Niakan^23^ | NA | III-IV | 20/30 (66.6%) | 20/30 (66.6%) | NA | NA | 4.8 ± 0.8 | 4.5 ± 1.2 | 12 |
| Piacenti^24^ | Yes (DNG 69.8% vs COC 88.4%) | I-IV | 7.8 ± 1.8 | 6.4 ± 2.5 | 8.4 ± 0.3 | 8.2 ± 0.2 | 7.4 ± 2.2 | 6.7 ± 2.2 | 24 |

DNG = Dienogest; COC = Combined Oral Contraceptives; NA = not available

*El Taha, Harada, Kashi and Piacenti et al reported pain as VAS score (mean ± SD); Niakan reported pelvic pain as number of patients answering “Yes” to a 3-point response question and dyspareunia as VAS score (mean ± SD)

**Table S3. Efficacy outcomes**

| Study ^Ref^ | Pelvic Pain after treatment* | | Dysmenorrhea after treatment* | | Dyspareunia after treatment * | | Treatment period (weeks) |
| --- | --- | --- | --- | --- | --- | --- | --- |
|  | DNG | COC | DNG | COC | DNG | COC |  |
| El Taha^20^ | 2.4 ± 2.1 | 3.3 ± 3.1 | 7/35 (20%) | 9.4/35 (26.9%) | 8.7/35 (25%) | 14.5/35 (41.7%) | 24 |
| Harada^21^ | 2.5 ± 2.3 | 4 ± 2.5 | NA | NA | NA | NA | 52 |
| Kashi^22^ | 3.2 ± 1.7 | 2.1 ± 2.3 | NA | NA | 2.6 ± 1.8 | 1.7 ± 1.4 | 24 |
| Niakan^23^ | 10/30 (33.3%) | 14/30 (46.6%) | NA | NA | 3.4 ± 0.9 | 2.4 ± 0.7 | 12 |
| Piacenti^24^ | 5.1 ± 2.0 | 4.3 ± 2.3 | NA | NA | 5.6 ± 2.5 | 4.7 ± 2.4 | 24 |

DNG = Dienogest; COC = Combined Oral Contraceptives; NA = not available

*El Taha, Harada, Kashi and Piacenti et al reported pain as VAS score (mean ± SD); Niakan reported pelvic pain as number of patients answering “Yes” to a 3-point response question and dyspareunia as VAS score (mean ± SD)

**Table S4. Tolerability data**

| Study ^Ref^ | Total drop out/discontinuation due to side effects | | Motivation of drop out | Abnormal vaginal bleeding§ | | Headache | | Reported weight gain | |
| --- | --- | --- | --- | --- | --- | --- | --- | --- | --- |
|  | DNG | COC |  | DNG | COC | DNG | COC | DNG | COC |
| El Taha^20^ * | 3/35 (8.5%) | 3/35 (8.5%) | DNG: irritability and weight gain (2 pt), irritability and spotting (1 pt); COC: prolonged bleeding (1 pt), irritability and weight gain (2 pt) | 21/31 (67.7%) | 29/32 (90.6%) | 10/31 (32.3%) | 19/32 (59.4%) | 3/31 (9.7%) | 11/32 (34.4%) |
| Harada^21^ | 0/53 (0%) | 18/130 (13.8%) | NA | NA | 48/130 (36.9%) | NA | 33/130 (25.4%) | NA | NA |
| Kashi^22^ | 1/30 (3.3%) | 1/30 (3.3%) | NA | 6/30 (20%) | 4/30 (13.3%) | 4/30 (13.3%) | 6/30 (20%) | NA | NA |
| Niakan^23^ | 1/30 (3.3%) | 1/30 (3.3%) | NA | 7/30 (23.3%) | 6/30 (20%) | 10/30 (33.3%) | 6/30 (20%) | NA | NA |
| Piacenti^24^ ** | 7/50 (14%)* | 7/50 (14%)* | DNG: weight gain (1 pt), vaginal bleeding (2 pt), vaginal bleeding and headache (1 pt), headache and abdominal swelling (1 pt), indication to surgery (1 pt), decreased libido (1 pt); COC: indication to surgery (2 pt), desire for pregnancy (2 pt), vaginal bleeding, headache and abdominal swelling (1 pt), weight gain (1 pt), other treatment (1 pt) | 11/43 (25.5%) | 10/43 (23.2%) | 12/43 (27.9%) | 8/43 (18.6%) | 6/43 (13.9%) | 9/43 (20.9%) |

§ Abnormal vaginal bleeding was described as follows: irregular or prolonged bleeding, infrequent bleeding or amenorrhea by El Taha; vaginal spotting by Harada, Tashi, Niakan; unexpected vaginal bleeding by Piacenti.

*The Authors reported as denominator the number of patients who actually received allocated intervention; data are reported according to the manuscript

** The Authors excluded from final statistical analysis patients dropping out the treatment before end of observation, reporting this data separately and not including those patients in final denominator

DNG = Dienogest; COC = Combined Oral Contraceptives; pt = patient; NA = not available

**Table S4. Tolerability data (continued)**

| Study | Decreased libido | | Breast pain | | Sleep disorder | | Fatigue | | Nausea/vomiting | |
| --- | --- | --- | --- | --- | --- | --- | --- | --- | --- | --- |
|  | DNG | COC | DNG | COC | DNG | COC | DNG | COC | DNG | COC |
| El Taha* | 1/31 (3.2%) | 3/32 (9.4%) | 6/31 (19.4%) | 15/32 (46.9%) | 3/31 (9.7%) | 9/32 (28.1%) | 3/31 (9.7%) | 9/32 (28.1%) | 5/31 (16.1%) | 17/32 (53.1%) |
| Harada | NA | NA | NA | 3/130 (2.3%) | NA | 4/130 (3.1%) | NA | NA | NA | 25/130 (19.2%) |
| Kashi | NA | NA | NA | NA | NA | NA | NA | NA | 4/30 (13.3%) | 6/30 (20%) |
| Niakan | NA | NA | NA | NA | NA | NA | NA | NA | 10/30 (33.3%) | 6/30 (20%) |
| Piacenti** | 7/43 (16.2%) | 5/43 (11.6%) | 5/43 (11.6%) | 5/43 (11.6%) | 5/43 (11.6%) | 8/43 (18.6%) | 9/43 (20.9%) | 10/43 (23.2%) | 2/43 (4.6%) | 6/43 (13.9%) |

*The Authors reported as denominator the number of patients who actually received allocated intervention; data are reported according to the manuscript

** The Authors excluded from final statistical analysis patients dropping out the treatment before end of observation, reporting this data separately and not including those patients in final denominator

DNG = Dienogest; COC = Combined Oral Contraceptives; NA = not available

**Table S4. Tolerability data (continued)**

| Study | Mood swings | | Abdominal discomfort | | Acne | | Depression | | Hair loss | |
| --- | --- | --- | --- | --- | --- | --- | --- | --- | --- | --- |
|  | DNG | COC | DNG | COC | DNG | COC | DNG | COC | DNG | COC |
| El Taha* | 14/31 (45.2%) | 24/32 (75%) | 5/31 (16.1%) | 12/32 (37.5%) | NA | NA | NA | NA | NA | NA |
| Harada | NA | NA | NA | 9/130 (6.9%) | NA | 6/130 (4.6%) | NA | NA | NA | NA |
| Kashi | NA | NA | NA | NA | NA | NA | NA | NA | 4/30 (13.3%) | 6/30 (20%) |
| Niakan | NA | NA | NA | NA | NA | NA | NA | NA | 10/30 (33.3%) | 6/30 (20%) |
| Piacenti** | 10/43 (23.2%) | 15/43 (34.8%) | 14/43 (32.5%) | 15/43 (34.8%) | 6/43 (13.9%) | 5/43 (11.6%) | 4/43 (9.3%) | 8/43 (18.6%) | 6/43 (13.9%) | 1/43 (2.3%) |

*The Authors reported as denominator the number of patients who actually received allocated intervention; data are reported according to the manuscript

** The Authors excluded from final statistical analysis patients dropping out the treatment before end of observation, reporting this data separately and not including those patients in final denominator

DNG = Dienogest; COC = Combined Oral Contraceptives; NA = not available

**Table S4. Tolerability data (continued)**

| Study | Hair loss | | Vaginal dryness | | water retention | | Hot flushes | | Meteorism | |
| --- | --- | --- | --- | --- | --- | --- | --- | --- | --- | --- |
|  | DNG | COC | DNG | COC | DNG | COC | DNG | COC | DNG | COC |
| El Taha* | NA | NA | NA | NA | NA | NA | NA | NA | NA | NA |
| Harada | NA | NA | NA | NA | NA | NA | NA | NA | NA | NA |
| Kashi | 4/30 (13.3%) | 6/30 (20%) | NA | NA | NA | NA | 4/30 (13.3%) | 6/30 (20%) | NA | NA |
| Niakan | 10/30 (33.3%) | 6/30 (20%) | NA | NA | NA | NA | 10/30 (33.3%) | 6/30 (20%) | NA | NA |
| Piacenti** | 6/43 (13.9%) | 1/43 (2.3%) | 4/43 (9.3%) | 2/43 (4.6%) | 9/43 (20.9%) | 13/43 (30.2%) | 4/43 (9.3%) | 4/43 (9.3%) | 6/43 (13.9%) | 11/43 (25.5%) |

*The Authors reported as denominator the number of patients who actually received allocated intervention; data are reported according to the manuscript

** The Authors excluded from final statistical analysis patients dropping out the treatment before end of observation, reporting this data separately and not including those patients in final denominator

DNG = Dienogest; COC = Combined Oral Contraceptives; NA = not available

**Table S4. Tolerability data (continued)**

| Study | Hand numbness | | Skin Dryness | | Treatment period (weeks) |
| --- | --- | --- | --- | --- | --- |
|  | DNG | COC | DNG | COC |  |
| El Taha* | NA | NA | NA | NA | 24 |
| Harada | NA | NA | NA | NA | 52 |
| Kashi | 1/30 (3.3%) | 4/30 (13.3%) | 1/30 (3.3%) | 1/30 (3.3%) | 24 |
| Niakan | 1/30 (3.3%) | 6/30 (20%) | 1/30 (3.3%) | 1/30 (3.3%) | 12 |
| Piacenti** | NA | NA | NA | NA | 24 |

*The Authors reported as denominator the number of patients who actually received allocated intervention; data are reported according to the manuscript

** The Authors excluded from final statistical analysis patients dropping out the treatment before end of observation, reporting this data separately and not including those patients in final denominator

DNG = Dienogest; COC = Combined Oral Contraceptives; NA = not available
